# GSearch: ultra-fast and scalable genome search by combining K-mer hashing with hierarchical navigable small world graphs

**DOI:** 10.1093/nar/gkae609

**Published:** 2024-07-16

**Authors:** Jianshu Zhao, Jean Pierre Both, Luis M Rodriguez-R, Konstantinos T Konstantinidis

**Affiliations:** Center for Bioinformatics and Computational Genomics, Georgia Institute of Technology, Atlanta, GA, USA; School of Biological Sciences, Georgia Institute of Technology, Atlanta, GA, USA; Université Paris-Saclay, CEA, List, Palaiseau, France; School of Civil and Environmental Engineering, Georgia Institute of Technology, Atlanta, GA, USA; Department of Microbiology, University of Innsbruck, Innsbruck, Austria; Digital Science Center (DiSC), University of Innsbruck, Innsbruck, Austria; Center for Bioinformatics and Computational Genomics, Georgia Institute of Technology, Atlanta, GA, USA; School of Biological Sciences, Georgia Institute of Technology, Atlanta, GA, USA; School of Civil and Environmental Engineering, Georgia Institute of Technology, Atlanta, GA, USA

## Abstract

Genome search and/or classification typically involves finding the best-match database (reference) genomes and has become increasingly challenging due to the growing number of available database genomes and the fact that traditional methods do not scale well with large databases. By combining k-mer hashing-based probabilistic data structures (i.e. ProbMinHash, SuperMinHash, Densified MinHash and SetSketch) to estimate genomic distance, with a graph based nearest neighbor search algorithm (Hierarchical Navigable Small World Graphs, or HNSW), we created a new data structure and developed an associated computer program, GSearch, that is orders of magnitude faster than alternative tools while maintaining high accuracy and low memory usage. For example, GSearch can search 8000 query genomes against all available microbial or viral genomes for their best matches (*n* = ∼318 000 or ∼3 000 000, respectively) within a few minutes on a personal laptop, using ∼6 GB of memory (2.5 GB via SetSketch). Notably, GSearch has an O(log(*N*)) time complexity and will scale well with billions of genomes based on a database splitting strategy. Further, GSearch implements a three-step search strategy depending on the degree of novelty of the query genomes to maximize specificity and sensitivity. Therefore, GSearch solves a major bottleneck of microbiome studies that require genome search and/or classification.

## Graphical Abstract

**Graphical Abstract:**
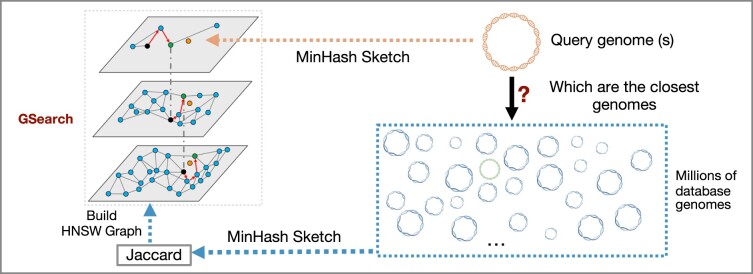

## Introduction

Classifying microbial species based on either universal marker genes (e.g. 16S or 18S rRNA genes) or entire genomes represents a re-occurring task in environmental and clinical microbiome studies. However, this task is challenging because the microbial genomes in nature are still severely under-sampled by the available genomes. For instance, the number of new genomes reported is still increasing. There are >10^12^ prokaryotic and fungal species in nature according to a recent estimation based on 16S rRNA gene or ITS (Internal Transcribed Spacer) analysis (1), and even more viral species, as the number of viral cells outnumbers that of prokaryotic cells by a about a factor of ten in most natural habitats (2). The number of total prokaryotic genomes has reached ∼318 000 in the newest release of the NCBI/RefSeq prokaryotic database (until February 2023), and >12 million in the latest IMG/VR4 database for viruses, representing 65 703 prokaryotic and 8.7 million viral distinct species if clustered at the 95% ANI (genome-average nucleotide identity) level (3,4). This has created a new challenge: an all-versus-all comparison strategy to search newly sequenced genomes against these large databases to find their best matches and/or classify them according to the matches has become impractical. Further, due to the recent improvements in metagenomic and single-cell sequencing technologies, it is now possible to recover hundreds, if not thousands, of genomes from environmental or clinical samples in a single study (5,6). Such studies have started to fill in the gap in the described diversity mentioned above but they have also exacerbated the database size problem. In addition to the searching strategy, the actual algorithm used to determine overall genetic relatedness (e.g. ANI or its approximations) between the query and the database genomes is critical. There are several ANI implementations available based on either BLAST (7), USEARCH (8) or MUMMER (9–12). While the traditional BLAST-based ANI, and the genome-aggregate average amino acid identity (AAI), have been proven to be highly precise and robust for genetic relatedness estimation across microbial and viral genomes (10,11,13,14), they take hours or even days of computational time when dealing with thousands of genomes. Recently, phylogenetic placement methods using a handful of universal genes (*n* ≈ 100) have become popular, but these methods can be memory demanding and slow (15,16), especially for a large number of or a few deep-branching (novel) query genomes. Further, the phylogenetic approach cannot be broadly applied to viral genomes, which lack universal genes. Moreover, universal genes, due to their essentiality are typically under stronger purifying selection and thus evolve slower than the genome average (17). This property makes universal genes appropriate for comparisons among distantly related genomes, e.g. to classify genomes belonging to a new class or a new phylum, but not the species and genus levels (15,18).

Faster and more memory efficient ANI estimation based on k-mer hashing and evolutionary models have been recently described in tools such as FastANI, Mash, Sourmash, Dashing and BinDash (19–23). These tools typically rely on probabilistic data structures (or sketching algorithms) to estimate genomic distance such as MinHash (a class of locality sensitive hashing) (24), FracMinHash (25), HyperLogLog (HLL) (26) or a combination of HLL and MinHash, called SetSketch (27). Importantly, MinHash and MinHash-like algorithms have been shown to provide an unbiased estimation of the Jaccard similarity $Jaccard( {A,B} ) = \frac{{| {A \cap B} |}}{{| {A \cup B} |}}$ between two genomes, an accurate proxy for ANI or mutation rate after appropriate transformations: $ANI\ or\ ( {1 - Mash} ) = 1 + \frac{1}{k}log\frac{{2*J}}{{1 + J}}$, where *J* is the Jaccard similarity and *k* is k-mer size, also known as the Mash equation. Note that the Jaccard similarity considers only unweighted k-mer presence/absence in a set (19). MinHash is more accurate than HyerLogLog, HyerLogLog++ or SetSketch(27) in estimating Jaccard similarity but less space efficient (21). However, Jaccard similarity (unweighted) estimated by MinHash-like sketching algorithms can be problematic for incomplete genomes (19,28) and genomes with extensive repeats (e.g. microbial eukaryotes) because this estimation does not consider the abundance of k-mers (k-mer multiplicity) and genome size. Weighted Jaccard-like indices such as those provided by ICWS and ProbMinHash have been recently developed to address this limitation (29,30) (see also Supplementary Note S1). The application of weighted (or unweighted) MinHash-like algorithms to the genome search problem (that is, to search a query genome against a genome database to find its closest relatives) can still be slow despite the algorithms themselves being fast because these algorithms are typically applied in a ‘brute force’ manner. That is, all query genomes are searched against all database genomes (i.e. ‘all versus all’), and thus computational time grows quadratically as the number of query and database genomes increase. More importantly, in the case of searching a database, the locality sensitive hashing property of those probabilistic data structures should be satisfied to ensure high recall or accuracy (31).

One of the most broadly used approaches for finding closely related information in a database while circumventing an all vs. all comparisons is the K-Nearest Neighbor Search (K-NNS). The K-NNS approach has been used, for instance, for 16S rRNA gene-based classification followed by a vote strategy (32). Approximate nearest neighbor search (ANNS) algorithms, such as locality-sensitive hashing (LSH) (33), k-dimension tree (34), random projection tree (35), k-graph (36) and proximity graph (37–39) have been recently used to greatly accelerate search processes with small loss in accuracy. Proximity graph, as implemented for example in the hierarchical navigable small world graph (HNSW), has been shown to be one of the fastest ANN search algorithms (40,41) with search time complexity ${\rm O}( {{\rm log}( N )} )$. HNSW incrementally builds a multi-layer structure consisting of a hierarchical set of proximity graphs (layers) for nested subsets of the stored elements. Then, through smart neighbor selection heuristics, inserting and searching the query elements in the proximity graphs can be very fast while preserving high accuracy, even for highly clustered data (38). Therefore, finding the closest genomes in a database can be substantially accelerated by combining two sub-linear algorithms while maintaining ANI/AAI accuracy: MinHash-like or HyperLogLog sketching algorithms for genomic distance estimation and HNSW for finding nearest neighbors. This is an idea that, to the best of our knowledge, has not been applied to genome search previously, despite its potential to greatly accelerate genome search.

Here, we describe GSearch (for Genome Search), a computer program that combines one of the most efficient nearest neighbor search approaches (HNSW) with MinHash-based or SetSketch-based estimates of genomic distances, and applied it to large collections of fungal, prokaryotic and viral genomes. Six MinHash-like algorithms are provided as part of GSearch to ensure the critical property of locality sensitivity: Densified MinHash (2 variants) (31,42), ProbMinHash (30), SuperMinHash (43) and SetSketch (2 variants) (27). Each of them provides distinct advantages in accuracy, speed and/or space requirement. Densified MinHash is by far the fastest algorithm due to the use of just one hash function. SuperMinHash is similar to classic MinHash but optimized in terms of accuracy to calculate simple Jaccard similarity, sacrificing speed. ProbMinHash (default) is based on shared k-mers, weighted by their abundance, and normalized by total k-mer count. Essentially, ProbMinHash computes the normalized weighted Jaccard-like similarity ${{J}_P}$ (Supplemenatary Note S1) between each pair of genomes. Accordingly, ProbMinHash can account for genome incompleteness and repeats (k-mer multiplicity) commonly found in eukaryotic and sometimes in prokaryotic genomes and is the default option (setting) in GSearch. SetSketch is a new data structure aiming at both space efficiency and speed, which fills the gap between MinHash and HyperLogLog. The Jaccard similarity calculated by any of the above methods is subsequently used as input to build the HNSW graph of the database genomes. Accordingly, the search of the query genome(s) against the graph database to find the nearest neighbors for classification purposes becomes an ultra-fast step and can be universally applied to all microbial genomes. The novelty of GSearch also includes a hierarchical pipeline that involves both nucleotide-level (when query genomes have close relatives at the species level) and amino-acid-level searching (when query genomes represent novel species), which provides high accuracy for query genomes regardless of their degree of novelty relative to the database genomes, as well as a database-splitting strategy that allows GSearch to scale up well to billions of database genomes.

## Materials and methods

**Figure 1.**
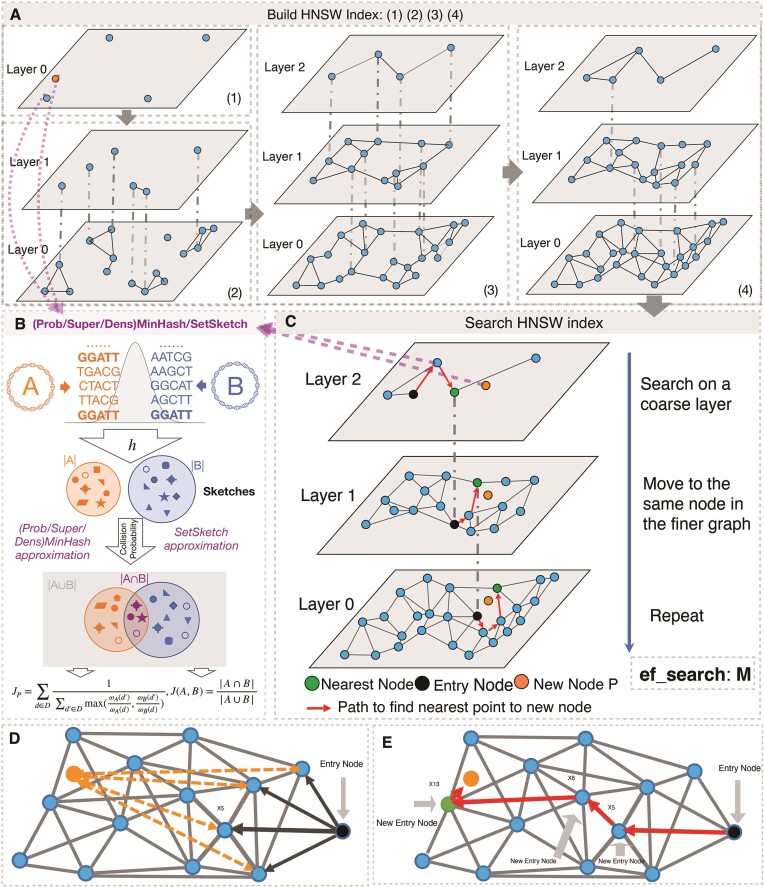
Schematic overview of GSearch building graph (**A**), graph searching (**C**) and (Super/Prob)MinHash/SetSketch distance estimation (**B**). (A) Overview of the HNSW building process. GSearch (tohnsw module) starts from a randomly chosen genome to gradually build the graph at Layer 0 (1) and gradually collapse genomes (dash grey line indicate newly clustered/collapsed representatives compare to previous step) into Layer 1 (2) and layer 2 (3) as more genomes are being incorporated in Layer 0 until all genomes in database are inserted into layer 0 (4). When inserting new genomes in the graph (database), GSearch essentially searches in a partially built graph to find nearest genomes, based on MinHash-like/SetSketch distance values among the genomes; see (**C**) for details. After the required number of nearest neighbors are found for each inserted genome, a reverse update step is performed to update neighbor list of all nodes in the graph. (**B**) Overview of (Super/Prob)MinHash/SetSketch algorithms to calculate *J_p_* and *J*, where *h* is the hash function to hash k-mers from two genomes (orange and blue) and store the hashed values as sketches (shapes in the two big circles). ProbMinHash calculates *J_p_* from shared sketches while SuperMinHash/Densified MinHash and SetSketch calculate J from shared sketches. (**C**) To search/identify a new genome P (orange) against the graph (request module), starting from an entry node (black, random or inherited from layer above it, depending on whether it is the top layer or not), GSearch finds the closest connected neighbor of the entry node (black) to the new node P to be searched (orange) by calculating the (Super/Prob/Den)MinHash/SetSketch distance of the new node P (orange) with all neighbors (blue) of entry node (black) and assigns the closes one (X5 in this case) as the new entry point (that is X5 will be the new entry node) (**D**). GSearch is then traverses in a greedy manner (i.e. update the entry point using the newly found closest connected neighbor of X5) until the nearest neighbors in the layer are found (specifically, the path goes from entry node to X5, X8 and X13) (**E**), and then goes to next layer. This process is repeated until the required number of nearest neighbors (N) are all found for the given new querying data point P and subsequently, reported to the user.

GSearch is composed of the following steps. Initially, the genetic relatedness among a collection of database genomes is determined based on the sketching algorithms Densified MinHash, ProbMinHash, SuperMinHash or SetSketch (30,31,42,43). These algorithms compute the normalized weighted Jaccard-like similarity *J*_p_ using the probminhash3a algorithm or the simple Jaccard similarity *J* using the SetSketch/SuperMinHash/Densified MinHash options, as implemented in the probminhash package. The normalized weighted Jaccard-like distance (1 – *J*_p_) or Jaccard distance (1 – *J*) is then used as input for building the HNSW graphs. Note that a distance computation is required only when that genome pair is required for graph building; thus, GSearch avoids all vs. all distance computations (Figure 1A). Genomes are subsequently recursively added as the nearest neighbors of each node in the built graph with the same distance computation procedure (Figure 1B). The built graph database and sketches from the MinHash-like algorithms are stored on the disk, including a graph file from HNSW as the main component of this new data structure. Each node/genome in the graph has a corresponding sketch in the sketching file (Figure 1B). Query genomes are then searched against the graph database after loading the database files in memory and subsequently, best neighbors are returned for classification (Figure 1C). In this process, the best neighbor(s) is also identified based on the smallest ProbMinHash distance (1 – *J*_p_) or Jaccard distance (1 – *J*) obtained. The output can then be transformed into ANI/AAI values with a separate program. The details of the whole Rust parallel implementation can be found in Supplementary Figure S8. Parallelization efficiency and memory usage of database building and searching can be found in Supplementary Figures S9 and S10. There are three modules in total: tohnsw, add and request. The tohnsw module builds the graph by gradually inserting genomes into the graph. The request module queries the graph database built in the tohnsw step. The add module adds new genomes to an existing HNSW database. Each of the three modules operates in parallel for high performance (see Supplementary Note S6). GSearch was implemented in the Rust programming language and can be found here: https://github.com/jean-pierreBoth/gsearch.

### Implementation of sketching algorithms and benchmarking

A detailed description of the differences between ProbMinHash and traditional MinHash can be found in the Supplementary Materials and Methods. We reimplemented the ProbMinHash algorithm to estimate genomic relatedness between any two genomes based on normalized weighted Jaccard-like distance 1 – *J*_p_ according to the original ProbMinHash paper (30) (Supplementary Note S1). The MSE (Mean Standard Error) of ProbMinHash is *J*_p_(1 – *J*_p_)/*m*, where *m* is the sketch size or number of registers, similar to that of classic Jaccard similarity *J*(*1*– *J*)/*m*. Essentially, when objects/k-mers are hashed, they represent probability distributions (relative frequency of k-mers after normalization), and *J*_p_ is a natural extension of *J* with Pareto optimality for estimating distance among various genomes (44). The Rust reimplementation of ProbMinHash, and other related MinHash-like algorithms, can be found at: https://github.com/jean-pierreBoth/probminhash. We relied on version 0.1.10 of probminhash package for this study. There are 11 different MinHash-like algorithms in this package (all are metric since *J* and *J_p_* are metric): One Permutation MinHash with Optimal Densification, Faster Densification, SuperMinHash, ProbMinHash1, ProbMinHash1a, ProbMinHash2, ProbMinHash3, ProbMinHash3a, ProbMinHash4, Order MinHash and SetSketch (locality sensitive hashing estimator and Joint Maximum Likelihood Estimator), with 6 of them used in GSearch (ProbMinHash3a (default), optimal densification, faster densification, SuperMinHash, SetSketch LSH and JMLE). Details of the locality sensitive hashing and JMLE implementation for SetSketch can be found in the Supplementary Methods and Materials. Specifically, when Jaccard similarity is smaller than 0.01 for queries with the best neighbors, LSH estimator in SetSketch is less accurate (Supplementary Figure S7). We therefore use JMLE estimator instead of LSH since it is more accurate for small Jaccard similarity values.

To benchmark ProbMinHash against Mash, Dashing v1, Dashing v2, Sourmash and BinDash, all tools were run with the same sketch size (*s* = 12 000) and k-mer size (*k* = 16) for bacterial genomes at the nucleotide level, and k-mer size (*k* = 7) at the amino acid level, for both database building and searching. For fungal genomes, a larger sketch size (*s* = 48 000) and k-mer size (*k* = 21) were used due to the much larger genome size. Further details on the rationale for choosing these k-mer sizes can be found in Supplementary Note S2. For convenient comparison of GSearch results against those of the Mash and ANI based methods, we performed the same transformation of Mash distance from normalized weighted Jaccard-like similarity *J*_p_ to ProbMASH-ANI as a proxy of ANI using the equation: $ProbMASH\_ANI = 1 + \frac{1}{k}log\frac{{2*{{J}_p}}}{{1 + {{J}_p}}}$. Details on how each software was run for the benchmarking can be found in Supplementary File S3.

### Hierarchical navigable small world graphs (HNSW)

Generally, the framework of HNSW can be summarized in the following two steps: (i) build a HNSW graph where each node represents a database vector (or sketch vectors of genome profiles in our case, Figure 1B). Each database vector will connect with a few of its neighbors while maintaining small world property in each layer of HNSW. (ii) Given a query vector, perform a greedy search on the HNSW graph by comparing the query vector with database vectors under the searching measures (in our case, 1 – *J*_p_ or 1 – *J*). Then, the most similar candidates are returned as outputs. The key task for these two-step methods is to construct a high-quality HNSW graph, which provides a proper balance between the searching efficiency and effectiveness. To guarantee the searching efficiency, the degree (number of maximum allowed neighbors, denoted as *M*) of each node is usually restricted to a small number (normally 20–200) while the effectiveness is ensured by a large width of search for neighbors during insertion (denoted as ef_construct; higher than 1000). The large ef_construct number increases the chance to find the best M neighbors by increasing the diversity of neighbors that can be retained. The graph is subsequently collapsed into hierarchical layers following an exponential decay probability. Building the graph (i.e. inserting nodes into the graph) and searching a query against the graph follow the same greedy search procedure except that there is an extra step of reverse updating of neighbors list for each vector when inserting database vectors (genomes), one by one, into the existing graph until all genomes are inserted (Figure 1A). For building, after searches are finished at the bottom layer for each inserted element, a reverse update step will be performed to update the neighbor list of each node in the existing graph, while for searching/querying against the graph this step is not needed. The overall database building time complexity is O(N*log(*N*)), where *N* is the number of nodes in the graph. The first phase of the searching process starts from the top layer by greedily traversing the graph to find maximum *M* closest neighbors to the new node element P in the layer by doing *ef*_construct times search (Figure 1C). After that, the algorithm continues the search from the next layer using the closest neighbor found from the previous layer as entry point, and the process repeats until it reaches the bottom layer. Closest neighbors at each layer are found by a greedy and heuristic search algorithm (Figure 1D and E). For searching, since there is no need to reverse update the best neighbor list for each node in the graph, time complexity is O(log(*N*)) as explained above (see also detailed complexity analysis on Supplementary Note S3). Theoretical guarantee of graph-based algorithms can be found in Supplementary Note S5. We reimplemented the original hnswlib library written in C++ using the Rust programming language for memory safety, efficient parallelism and speed. We also implemented memory map in the newest version, a feature not in the original C++ library that is useful for billions of data points or genomes. The implementation can be found at https://github.com/jean-pierreBoth/hnswlib-rs. Version 0.1.19 was used in this study. Speed, accuracy and scalability benchmark of this package against the original implementation using standard datasets can be found in Supplementary Tables S7–S10. In general, our implementation is as competitive as the original implementation in all three aspects.

### Prokaryotic genome search pipeline

For building the whole-genome amino-acid graph, we used *k* = 7 to have the best specificity without compromising sensitivity, which is also consistent with previous results on classification of amino acid sequences based on k-mers (45). For building the graph based on universal gene set, we used *k* = 5 because of the much smaller total amino acid space of universal genes. For further details on the range of k-mer to use for bacteria genome and proteome, and viral/fungal genome and proteome, see Supplemental Note S2.

The proteome of each genome was predicted by FragGeneScanRs v0.0.1 for performance purposes as opposed to Prodigal (46) software despite small loss in precision (47) (Supplementary Table S11). Hmmsearch in the hmmer (v3.3.2) (48) software was used to extract the universal gene set for bacteria and archaea genomes. Note that for viral genomes, this last step was not implemented because there are no universal single copy genes for viral genomes. Evaluation of the speed and memory requirements for all steps mentioned above were performed on a RHEL (Red Hat Enterprise Linux) v7.9 with 2.70 GHz Intel(R) Xeon(R) Gold 6226 CPU. Unless noted otherwise, all 24 threads of the node were available by default.

In order to directly compare GSearch results with GTDB-Tk v1.3.0, we used the top-10 matches provided by GSearch for each query genome against GTDB r207, and perform the evaluation as follows. If the best match (top 1) had ANI ≥ 95% with a database genome, the query was (manually) identified as the same species as the match and this result was compared to GTDB-Tk v1.3.0′s classification for the same query (i.e. whether or not it was assigned to the same species). Similarly, for genomes with at least 5 matches out of 10 with AAI ≥ 65% to the same genus, the query was identified as the same genus (but new/novel species) as these matches. Finally, for the remaining query genomes with top 5 best matches out of 10 with AAI ≥ 52%, the query was identified as the same family (but new/novel genus and species) as these matches, otherwise the genome was considered unclassified at the family level.

### Distributed implementation and database splitting

To accommodate the increasing number of microbial genomes that has become available at an exponential pace in recent years, and will soon surpass 1 million, we provide an option to randomly split the database into a given number of pieces and build a graph database separately for each piece. In the end, all best neighbors returned from each piece are pooled and sorted by distance to have a new best K neighbor collection returned to the user for each query genome. It has been proven that in terms of requesting top K best neighbors, the database split strategy is equivalent to non-split database strategy if the requested best neighbors for each database piece is larger than or equal to K (38,39). The database splitting and request can be done sequentially, on a single node, when multi-node support is not available. In theory, a large database can be split into any number of pieces. In practice, a reasonable way to decide on the number of database pieces to use is so that memory requirement for each piece is equal or smaller than the total memory of host machine.

### Species database and testing genomes for benchmarking and recall

The GTDB version 207 and the entire NCBI/RefSeq prokaryotic genomes (as of Feb. 2023) were used to build the database for bacterial and archaeal genomes. The IMGVR database version 4, with species representatives at a $ \ge$95% ANI, was used to build the database for viruses (a total of ∼3 million). For fungal genomes, all genomes downloaded from the MycoCosm project (as of Jan. 2022) were used (49). The amino acid sequences of predicted genes on the genomes were obtained using FragGeneScanRs for bacteria/archaea and GeneMark-ES version 2 for fungi (50).

To test the accuracy of GSearch, we specifically chose genomes that are not included in the GTDB database (the database that was used for graph building). In particular, the bacterial/archaeal genomes, mostly MAGs from activated sludge samples, reported by Ye *et al.* (51) were used. We randomly selected 1000 MAGs from the Ye's collection and use them as query genomes to test the accuracy of GSearch. Tara Ocean MAGs (total 8466 MAGs) (52) were also used for accuracy test.

### Recall of AAI-, ANI- and MinHash-based nearest neighbor searching for bacteria/archaea, fungi and viral genomes

To benchmark GSearch performance compared to traditional ANI/AAI- and/or MinHash-based tools, we ran brute-force ANI/AAI searches of the same query genomes against the (same) reference databases and assess if GSearch return the same best match as these traditional methods. We used BLAST-based ANI (http://enve-omics.ce.gatech.edu/ani/, ANI calculator) for bacteria and archaea as well as for viral collections, MUMMER-ANI for fungi, and BLAST-based AAI in all databases. We also use OrthoANI as an additional ANI gold standard and it showed the same ground truth (i.e. same best match) as ANI calculator (Supplementary Table S20). We also performed additional benchmarks using FastANI as the truth since FastANI correlates perfectly with BLAST-ANI (Supplementary Figure S13). For the assessment, we used the well-established average recall as the accuracy measurement (53). Given a query genome, GSearch is expected to return K best genomes. We examined how many genomes in this returned set were among the true K nearest neighbors (NN) of the same query found by the reference brute-force ANI/AAI. Suppose the returned set of K genomes given a query is R’ and the true K nearest neighbors set of the query (from BLAST-ANI/AAI) is R, the recall is defined as: $recall( {R^{\prime}} ) = \frac{{| {R^{\prime} \cap R} |}}{{| R |}}$. Then, the average recall represents the mean recall for all query genomes. We compare the accuracies of different algorithms by requiring different numbers of nearest neighbors (NN) of each query genome, including 10-NN and 5-NN. Since biological species databases are generally sparse due to undersampling of natural diversity and the existence of natural gaps in diversity among species, a larger top K NN (e.g. 100) used in standard ANN benchmark experiments will offer little, if any biological advantage, especially when the query genomes are relatively novel, e.g. a new family compared to database genomes. Therefore, we use top 5-NN and 10-NN. Further, if the genomic Jaccard distance (1 – *J*_p_ or 1 – *J*) of query to some of the top 10 or top 5 neighbors found by GSearch at the nucleotide level was larger than 0.9850 for bacterial genomes, these matches were filtered out and not used in estimation of recall because we have shown that above this threshold, k-mer based MinHash methods at nucleotide level will lose accuracy and this is not related to the HNSW search itself (e.g. 8 out of 10 are kept, so top 8 in R’ is compared with top 8 in R). At the proteome level, accuracy was calculated following a similar procedure with the threshold value being 0.9720 (switching to the universal gene graph above this threshold). For viral genomes, the threshold was 0.9800. These thresholds were chosen based on correlation analysis between the GSearch genomic distance and BLAST-based ANI or AAI. Details about how each piece of software was run can be found in Supplementary File S3.

## Results

### ProbMinHash is a robust metric of genome relatedness for prokaryotic genomes

Correlations between ProbMASH-ANI (we called it ProbMASH, after transformation from ProbMinHash similarity (*J*_p_), see Materials and Methods for details) and ANI determined by FastANI or Mash-ANI showed that ProbMASH-ANI is robust and slightly better than Mash for determining distances among bacterial genomes related at ∼78% ANI, or above (Spearman rho = 0.9643 and 0.9640, respectively, *P*< 0.001, Supplementary Figure S1a and b). For moderately related genomes, for which ANI based on nucleotide k-mer is known to lose accuracy, ProbMASH-AAI based on amino acid k-mer was robust compared to BLAST-based average amino acid identity (AAI), especially between genomes showing 95% > AAI > 52% (Spearman rho = 0.90, *P*< 0.01, Supplementary Figure S2a and b). Below ∼52% AAI, both ProbMASH-ANI and Mash-ANI lose accuracy compared to AAI. However, AAI of just universal genes provides a robust measurement of genetic relatedness at this level of distantly related genomes, and we show here that ProbMASH-AAI for this set of universal genes is also robust (Spearman rho = 0.9390, *P*< 0.001, Supplementary Figure S3). Thus, for query genomes with distant relatives in the database (i.e. deep-branching, novel genomes), for which their closest matching genome in the database is related at the order level or higher, restricting the search to the universal genes can provide robust classifications (15). Accordingly, GSearch implements a three-step classification process, depending on the degree of novelty of the query genome against the database genomes, using the ANI and AAI thresholds mentioned above (see also Figure 3F). This strategy and its accuracy are discussed further below. Similarly, One Permutation MinHash with Optimal or Faster Densification (Densifed MinHash) was also shown to be highly accurate in predicting ANI/AAI (Supplementary Figure S11).

### Comparisons with other sequence search algorithms

We compared GSearch with other general-purpose sequence (e.g. Sequence Bloom Tree, COBS) or MinHash-based genome search algorithms, focusing on time complexity (big O notation) of each algorithm. GSearch was clearly the fastest genome search algorithm, with a *O(log(N*)) complexity (Table 1). Other general-purpose sequence search algorithms were either sublinear under certain assumptions or not practical for large genome collections. Also, none of the general-purpose sequence or genome search algorithms had been previously benchmarked for their estimation or prediction of whole-genome ANI relatedness; hence, their accuracy remains untested.

**Table 1.** Bioinformatics algorithms for sequence/genome searching and their comparisons. *S* represents a document/genome. *N* is the total number of documents/genomes. Us∈S |*s*| represents the total number of terms in *N* documents/genomes and ∑*s*∈*S* |*s*| is total unique terms/k-mer. MPH is minimal Perfect Hashing. For the Inverted Index size, the extra log (*N*) comes from the bit precision document IDs. For SBTs, log(*N*) is the height of the tree, and for Bloom Filters at each level is ${\mathrm O}\,\left( {\sum s \in S{\mathrm{ }}| s |} \right)$ in total. MinHash time and space complexity was based on *k* hash functions for *N* sets, each set has d’ non-zero element. Note that the most recent B-bit MinHash with optimal densification can further improve to O(N*(*d*’+*k*)). O(*v*dlog(*N*)) for GSearch is O(log(*N*)) in practice since *v* and *d* are small and single pair genome comparison is a constant (Supplementary Note 3). τ depends on the user-specified parameter and is generally <0.01 in practice

	Query time	Space	Recall benchmark (Biologically)	Comments
Inverted Index^1^	$O( 1 )$	$Best:log( N )\mathop {\cap}\limits_{s \in S} | s |$	Only for document search/retrieval, could be applied to genomic/sequence search	Long construction time; impractical for bigger datasets; best case needs MPH and a known k-mer (term) distribution
BIGSCI/COBS^2,3^	$O( N )$	$\mathop \sum \limits_{s \in S} | s |$	A hybrid between an inverted index and Bloom filters (COBS), high false positive rate, no benchmark with mutation rate by ANI/Mash	Query time is linear in N, small index size
Sequence Bloom Tree^4^	$Best:O( {\log ( N )} ),\ Worse:O( N )$	$log( N )\mathop \sum \limits_{s \in S} | s |$	Given the k-mers from a query sequence, the task is to determine which of the N documents contain all the k-mers present in the query; no benchmark with mutation rate by ANI/Mash	Sequential query process is bottleneck; designed for sequential implementation
RAMBO^5^	$O( {\sqrt N *log( N )} )$	$\Gamma log( N )\mathop \sum \limits_{s \in S} | s |$	Similar to SBT, finding which of the N documents/genomes contain all the k-mers present in the query, no benchmark with ANI/Mash	Only for $\Gamma$ < 1, query time is sub-linear
MinHash^6^	$O( {N*d^{\prime}*k} )$	$O( {N*k} )$	Average Nucleotide Identity (ANI) or mutation rate via Mash distance	Query time is linear in N
GSearch (MinHash-like + HNSW)	$O( {vdlog( N )} )$	$O( {( {1 + \tau } )*N*d + k*N} )$	Average Nucleotide Identity (ANI) via Mash-like mutation rate/index	Long database construction time $O( {N*log( N )} )$, but users are free from construction.
FLINNG^7^	$O( {lo{{g}^2}( {\frac{1}{\delta }} )lo{{g}^3}( N ){{N}^{\frac{1}{2} + \gamma }}\ } )$	${{N}^{\frac{3}{2}}}lo{{g}^2}( N )$	No Benchmark with Average Nucleotide Identity (ANI) via Mash-like mutation rate/index, only 15% of RefSeq genome meet the $\gamma$-stable criteria	The $\gamma$-stable query condition is a relatively strong requirement for the query. Limitation: works for queries for which the neighbors are all above a (relatively high) similarity threshold to the query

^1^Croft *et al.* (84), ^2^Bradley *et al.* (85), ^3^Bingmann *et al.* (86), ^4^Solomon and Kingsford (87), ^5^Gupta *et al.* (88), ^6^Broder (24), ^7^Engels *et al.* (89).

We then compared available MinHash-like sketching algorithms in terms of their time complexity (big O notation), space (memory), and accuracy in estimating the Jaccard similarity (Supplementary Tables S1 and S2), as well as other important properties for large scale applications such as mergeability (whether sketching parts of the dataset individually and subsequently merge them is equivalent to sketching the entire dataset). SuperMinHash and ProbMinHash were fast and mergeable MinHash-like algorithms, slower than Densifed MinHash (Supplementary Table S1). The latter algorithm, however, is not mergeable, thus difficult to use in distributed computational environments. HyperLogLog-like algorithms are significantly more space-efficient albeit slightly slower and less accurate. For variance, we analyzed the rooted mean squared error (RMSE) with respect to true Jaccard similarity. MinHash-like algorithms such as ProbMinHash, SuperMinHash and Densified MinHash had the smallest theoretical variance for the same m (the number of registers used for sketching; we use *m* = 12 000), followed by SetSketch (as *b*$ \to$1) (Supplementary Table S2). Our actual implementation of Densified MinHash has similar RMSE to classic MinHash (Supplementary Figure S12), with estimated ANI correlating well with that of Mash (classic MinHash) (Supplementary Figure S11). SetSketch was also space-efficient and similar to HyperLogLog in terms of space, speed and accuracy. HyperLogLog sketch estimators such as those implemented in Dashing had the largest RMSE for estimating the cardinality of sets (followed by inclusion-exclusion rule to estimated Jaccard similarity, Supplementary Note S1), consistent with experimental results when searching query genomes against database (Supplementary Tables S14 and S15). RMSE of Dashing was especially large for small Jaccard similarity (e.g. 75% to 78% ANI, corresponding to 0.009 and 0.015 Jaccard similarity, respectively; Supplementary Tables S14 and S15).

### Graph building and speed of search against the reference prokaryotic genomes

**Figure 2.**
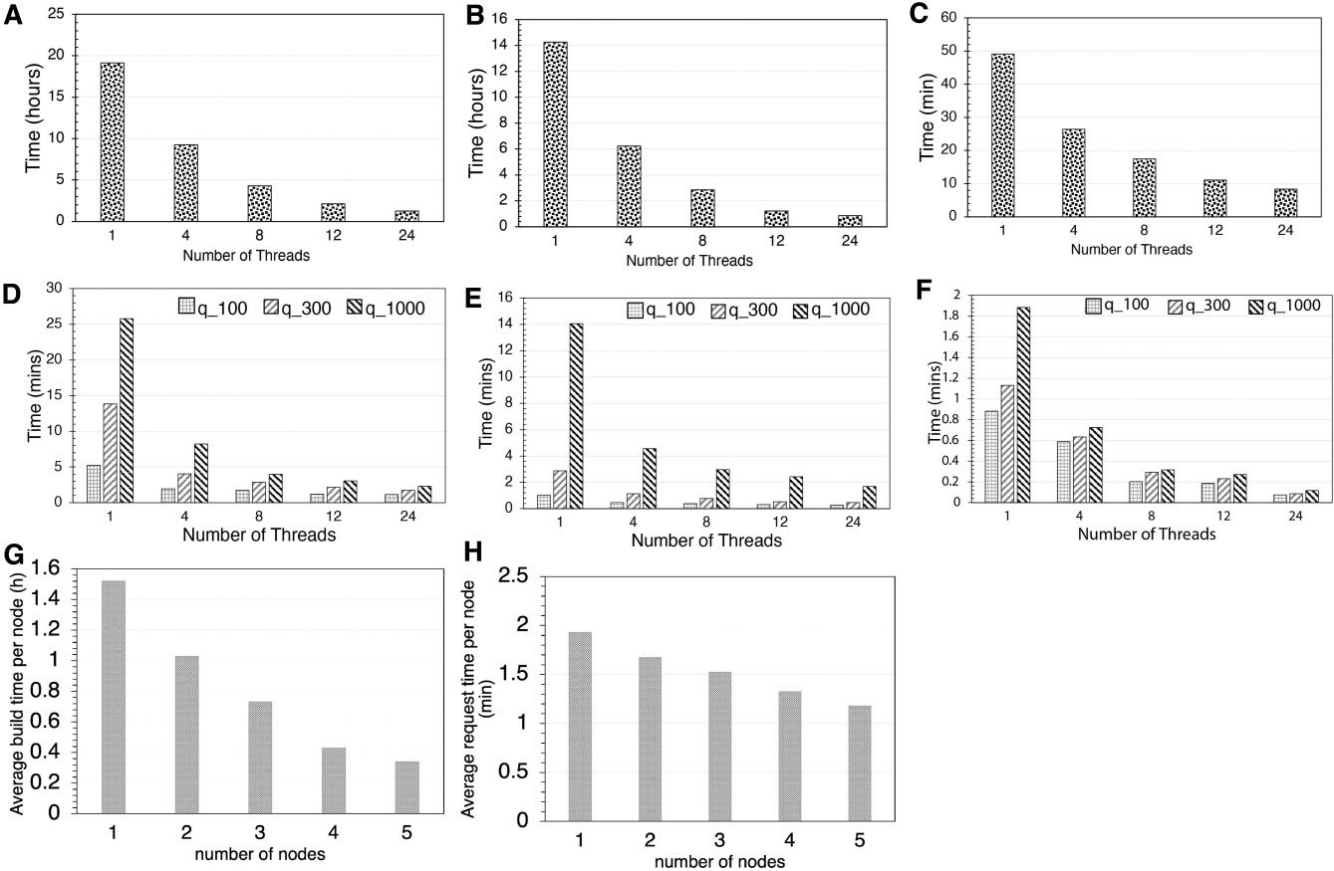
Performance and scalability of database building (tohnsw) and searching (request) steps as the number of threads or nodes increases. Upper panel shows total wall time (y axis) for building the GTDB v207 (65703 genomes) at the nucleotide level (**A**), whole-genome proteome (amino acid level) (**B**) and universal gene set proteome (**C**) reference graphs (or databases). Middle panel shows searching performance and scalability against number of threads used for different sets of query genomes at nucleotide (**D**), amino acid level (**E**) and universal protein level (**F**). Specifically, 100, 300 and 1000 query genomes (figure key) were used. All tests were run on a 24-thread Intel **(R)** Xeon (R) Gold 6226 processor, with 40GB memory available (**G**). Database build time vs number of nodes or number of database pieces (maximum 5) for the split strategy, and request/search time of 100 bacterial genomes (nt) against the split databases (**H**). Each node was responsible for running a piece of the full database. Average time was calculated by averaging total time across all nodes for (G) and (H). The entire NCBI/RefSeq database (318K genomes) were used for testing the split strategy. For each node, 24 threads were used for building and searching steps.

To build the database graph for the entire GTDB v207 prokaryotic species database at the nucleotide level (65703 unique, non-redundant prokaryotic genomes at the 95% ANI level), the tohnsw module (database build subcommand of GSearch) took 1.3 h on a 24-thread computing node and scaled moderately well with increasing number of threads (0.27 h using 128 threads) (Figure 2A). It took 0.7 h using Densified MinHash for building and is slower using SuperMinHash (4.7 h) and SetSketch (2.4 h) (Supplementary Table S18). Maximum memory (RAM) required for the building step was 15.3 GB. The total size of the written database files on disk was 3.0 GB. There are 3 layers for the resulting graph, with 65 180, 519 and 4 genomes for layers 0, 1 and 2, respectively. The searching of query genomes against the GTDB database graph, which represented previously known as well as novel species of eight bacterial phyla (see Materials and methods for details on query genome selection), requesting best 50 neighbors for 1000 query genomes, took 2.3 min (database loading was 6 s) on a 24-thread machine and scaled well with increasing number of threads (Figure 2D). The memory requirement for the request (search) step was 3.0 GB for loading the entire database file in memory.

**Figure 3.**
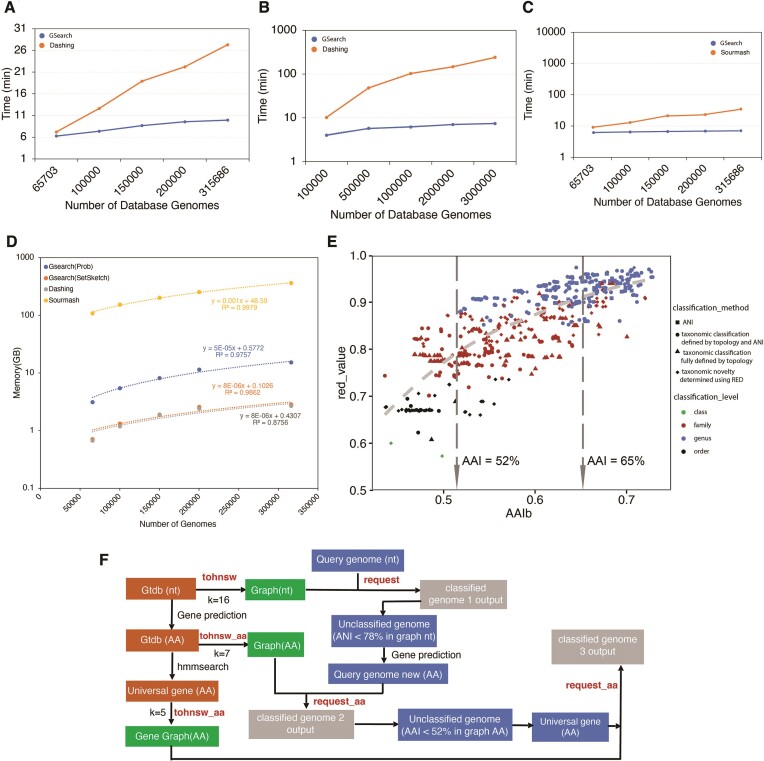
Running time, memory consumption and classification accuracy of GSearch against Dashing, Sourmash and BLAST-based ANI/AAI tools. (**A**) Running time of GSearch versus Dashing for searching 8466 query genomes against the RefSeq prokaryotic genome database as a function of the number of genomes used in the database (x-axis) at the nucleotide level. (**B**) Running time of GSearch (blue) versus Dashing (orange) for 10 000 query viral genomes against the IMG/VR v4 database at amino acid level. (**C**) Same as in (A) above but comparison is against Sourmash multisearch (orange). (**D**) Memory consumption of GSearch versus Dashing and Sourmash search. Search is to load database into memory, thus maximum memory is directly related to database size. Since Sourmash search is not parallelized, GNU parallel was used for process-level parallelism. Note that y-axis values are in log scale in panels (B)–(D). (**E**) Comparison of GSearch classification results with GTDB-Tk and Blastp-based AAI tools for moderate-to-distantly related query genomes based on the bacterial proteome database (e.g. ANI between the query genome and its best match in the database was lower than 78% for these genomes). Each point represents a comparison between two genomes, query and the best match found by GSearch, showing RED values generated by GTDB-tk (y-axis) versus Blastp-based AAI between these two genomes. The taxonomic rank that the query and the best database match share is shown (see figure key). Two vertical lines indicate Blastp-based AAI threshold for family and genus level classification threshold. Note that the best match was always the same genome between GSearch and all vs. all Blastp AAI, and the overall consistency between GSearch/AAI and GTDB-tk in identifying the same best database genomes for the same query genomes. (**F**) Overview of GSearch's 3-step pipeline for classifying prokaryotic genomes. Orange boxes denote steps that aim to prepare genome files, in different formats, for graph building while green boxes denote building steps of the graph database (in nucleotide or amino acid format). Blue boxes indicate input/query genomes to search against the database while grey boxes indicate classification output for each input. Two key steps of GSearch: tohnsw and request are used to build graph database and request (or search) new genomes against the database, respectively. Two thresholds are used in the pipeline to decide between whole nucleotide vs. whole-genome amino acid search and whole-genome amino acid vs. universal gene amino acid; that is, 78% ANI and 52% AAI, corresponding to Probminhash distance 0.9850 and 0.9375, respectively (see main text for details).

We also built a database of all NCBI/RefSeq prokaryotic genomes (∼318K genomes, 2 TB in size, in total), which took 4.1 h using 24 threads with maximum memory usage of ∼21 GB (1.2 h with 128 threads) and created a stored database file size of 15 GB. It took 1.4 h using Densified MinHash for building and was slower using SuperMinHash (27 h) and SetSketch (13 h) (Supplementary Table S19). Searching of 8466 query genomes against this RefSeq database took 9.33 min (Table 2, ProbMinHash) and ∼16 GB of memory, which was significantly better than alternative state-of-the-art tools for the same purposes such as skani, Dashing v1 and v2 and BinDash. For example, Dashing v1 or v2 and BinDash (brute force HyperLogLog or MinHash) took 21, 42 min and 41 min for the same task, respectively (Table 2). To evaluate the performance against Mash, Dashing, Sourmash or BinDash more fully, we increased the number of database genomes gradually from a subset of 65 703 to the full set of 315 686 genome while using the same number of query genomes (8466). We observed that GSearch follows a log fitting, consistent with the *O(log(N))* theoretical prediction, while Dashing, Sourmash, MASH and BinDash followed linear fitting (Figure 3A and C, Supplementary Figures S4a and b, S5a and b). For example, GSearch was 3–4 times faster than Dashing when the number of database genomes increased from 65 703 to 315 686 (Figure 3A), and 20–30 times faster with the 3 million reference virus genomes (Figure 3B, see also below). With Densified MinHash, GSearch can be even faster than this (Table 2).

**Table 2.** Comparisons of database size, running time for ProbMinHash, Densified MinHash, SuperMinHash and SetSketch with all other pieces of software

	Database size (indicates minimum memory required)	database loading time	Sketching & searching	Total Wall time
GSearch(ProbMinHash)	29GB (sketch + graph)	57.2 s	8 min 23 s	9 min 20 s
GSearch(Densified MinHash)	9.7GB	27.2 s	4 min 12s	4 min 40 s
GSearch(SuperMinHash)	25GB (sketch + graph)	41 s	24 min 13 s	24 min 54 s
GSearch(SetSketch)	2.5GB (sketch + graph)	9.1 s	12 min 14 s	12 min 23 s
Mash	19.8GB	1 min 11 s	182.1 min	183 min
Sourmash (branchwater)	7.9GB	∼30 s	64.3 min	65 min
Dashing 1	2.7GB (sketch)	∼61 s	20.5 min	21.5 min
Dashing 2 (ProbMinHash)	4.7GB (sketch)	∼61 s	47.5 min	48.5 min
skani^a^	30.1GB	-	74.42 min	74.42 min
BinDash	16.2GB (Sketch)	16 s	51.4 min	41.4 min

^a^For ∼8000 query genomes, skani total memory requirement is larger than 60GB, the largest among all pieces of software.

Results are based on searching 8466 query genomes against all NCBI/RefSeq genomes (∼318K) on a 24-thread machine. Times reported are average values from 3 runs.

To build the amino-acid-level graph for moderately novel query genomes, all GTDB v207 proteomes were predicted using FragGeneScanRs and subsequently, the predicted amino acid sequences were used for building the database graph. The graph building step took 1.4 h (Figure 2B) with a maximum memory requirement of 37.7 GB on a 24-thread node. It took 0.7 h using Densified MinHash and was slower using SuperMinHash (3.9 h) and SetSketch (2.7 h). The total size of the written database files on disk was 5.9 GB. With the SetSketch option, CPU time was slightly longer (2.7 h) but maximum memory (9.4 GB) and database size (0.5 GB) were substantially smaller, as anticipated (Figure 3D). There were 65 158, 543 and 2 genomes for layer 0, 1 and 2 respectively. The entire RefSeq genome database (∼318K) took 3.7 hours to build at the proteome level with maximum memory ∼30 GB. Dumped files size was 29 GB (2.5 GB with the SetSketch option). Requesting 50 neighbors for querying 1000 genomes at the amino-acid level took 1.52 min with a memory requirement of ∼6.0 GB (database loading 9 s; Figure 2E) for the GTDB database. Requesting 50 neighbors for 8466 query genomes against the NCBI/RefSeq proteome graph, took about 6.2 min (Supplementary Table S3). In comparison, Mash dist took 207.2 min against NCBI/RefSeq for the same task with 24 threads (no amino acid option is available for Dashing, skani and BinDash).

Finally, for most distantly related query genomes, the graph building for the universal gene set follows the same logic as the amino acid level graph mentioned above except for using a smaller k-mer size (*k* = 5) due to the smaller k-mer space of the ∼120 universal genes used in this graph vs. the whole-genome previously. It took 7.76 min to build the GTDB database graph (Figure 2C), and 7 s to request 50 neighbors for 1000 queries on a 24 threads node against the database (Figure 2F, Supplementary Table S5). Building the entire NCBI/RefSeq database took about 27 min while requesting 1000 queries against it took about 0.445 min (Supplementary Table S3).

### Searching accuracy and speed for prokaryotic genomes

The accuracy of GSearch and other tools in finding the best matching genomes among the database genomes was evaluated by comparing their results against those identified by traditional alignment-based methods, namely BLAST-based ANI for query genomes with close relatives in the databases and BLAST-based AAI for more divergent query genomes. The query genomes originated from two datasets, the Tara Ocean (8466) and the collection put together by Ye *et al.* For the genomes in these two datasets that had closely related genomes in the RefSeq database (showing > 78% ANI, 6992 and 906 query genomes, respectively), we observed an average top 10 recall of 96.2% and 95.1%, respectively (similar results were obtained for top-5 matching genomes; Table 3). For query genomes with no closely related database genomes showing higher than 78% ANI, 1474 and 91 genomes from the two datasets, respectively, recall was significantly lower, around 60% or below (Table 3). However, when we search these genomes at the proteome level using BLAST-based AAI as the reference standard for calculating recall, the recall value increased to 96.9% and 95.2%, respectively (Table 3). There were 327 and 25 genomes, respectively, that, even at the proteome level, had no related genome in the RefSeq database showing higher than 52% AAI, and these genomes accounted for most of differences with the top-10 matching genomes by BLAST-based AAI. These genomes apparently reflect the fact that the public genome databases are still sparse and do not cover well all biological diversity. When we searched these deep-branching (highly novel) genomes at the universal gene proteome level, using the universal gene AAI as the reference standard, the average top-10 recall value was 95.5% and 94.6%, respectively (Table 3). We also examined the results obtained by BinDash, Dashing v1 and v2 and Sourmash for the same query genomes. We found that Sourmash was as accurate as GSearch but much slower, while Dashing is less accurate than GSearch and Sourmash or BinDash, especially when the closest database genomes showed less than 80% ANI to the query genomes (moderately or distantly related) (Supplementary Tables S13 and S14). The results were similar independent of the estimator used in Dashing (e.g. MLE or JMLE) (Supplementary Table S15), consistent with theoretical predictions (Supplementary Table S2). GSearch's Densified MinHash, SetSketch and SuperMinHash options showed similar results with the default ProbMinHash for the same query genomes (Supplementary Table S16). We chose the 78% ANI and 52% AAI as thresholds to switch between whole-genome nucleotide vs. amino acid (proteome) search and whole-genome proteome vs. universal gene only, respectively (Figure 3f). Detailed nucleotide-level accuracy results for all tools based on a single query genome (not average recall across all query genomes as shown above) can be found in the Supplementary Tables S13–S15. Proteome-level results for this single query genome can be found in Supplementary Table S17, which shows that GSearch is as accurate as Sourmash and MASH.

**Table 3.** Recall of GSearch for two query genome datasets containing various levels of novel genomes relative to the genomes in the reference database

Dataset	Dataset size (# of genomes)	level	Recall (top 5)	Recall (top 10)
**Tara (with best matches in database)** ^a^	**6992**	**nucleotide**	**98.3%**	**96.2%**
Tara (without best matches in database)	1474	nucleotide	62.4%	43.1%
**Tara (with best matches in database)** ^b^	**1147**	**proteome**	**97.2%**	**96.9%**
Tara (without best matches in database)	327	proteome	65.0%	50.8%
**Tara (with best matches in database)** ^c^	**327**	**universal**	**96.4%**	**95.5%**
Ye (with best matches in database)^a^	906	nucleotide	97.7%	95.1%
**Ye (without best matches in database)**	**91**	**nucleotide**	**54.8%**	**48.5%**
Ye (with best matches in database)^b^	66	proteome	96.3%	95.2%
**Ye (without best matches in database)**	**25**	**proteome**	**76.0%**	**55.5%**
Ye (with best matches in database)^c^	25	universal	95.7%	94.6%

^a^Defind as query genomes that had best match in the database better than 78% ANI. ^b^Defined as query genomes that had best match in the database better than 52% AAI. ^c^defined as query genomes that had best match in the database better than 55% AAI based on universal genes.

The query genome datasets are the same as those used in Table 2. BLAST-ANI, BLAST-AAI and universal gene BLAST-AAI were used as the reference results (or standards) to compare against. Recall is the average across all query genomes.

We also evaluated the effect of genome completeness on the accuracy of GSearch given that bacterial genomes recovered from environmental metagenomic surveys are frequently incomplete. We found that for genome completeness higher than 50%, the accuracy/recall in the top 10 best matches was higher than 80% and decreased considerably below this completeness level (Supplementary Table S12). Hence, GSearch analysis is not recommended for genome completeness less than 50% due to the normalization step in ProbMinHash.

### Graph database building and searching accuracy for viral and fungal genomes

Graph building and requesting for viral genomes is not effective at the nucleotide level because many viral genera are genetically too distant from each other and do not have close relatives in the public genomic database; that is, the database is too sparse. Therefore, we built only an amino acid level graph for viral genomes, using all genes in the genome due to the lack of universal genes for viral genomes. Database building took 13.89 h for all ∼3 million IMG/VR4 genomes on a 24-thread node, and graph file size on disk was 15.8 GB (Supplementary Figure S6a). Requesting 1000 top neighbors (10 are used for accuracy evaluation) scaled well with increasing number of threads and took 3.63 min (database loading took an additional 1.1 min) using 24 threads (Supplementary Figure S6b). The top-5 neighbors for 1000 query phage genomes were highly overlapping (98.32% recall; Supplementary Table S6) compared number of genomes, GSearch is about 20X faster than Mash and Dashing in finding the top-5 neighbors (Figure 3B, Supplementary Tables S6). We also compared GSearch with a new database building method, called PhageCloud, which relies on manually curated genome labels (e.g. environmental source) for graph database building using the Neo4j software and Dashing for genetic distance computation. Since PhageCloud provides a web implementation with one genome query at a time, we limited the search to one viral query genome against the same database used by PhageCloud i.e. the Gut Phage Database. It took 37 s to find the two best matches with PhageCloud while GSearch took 15 s (database loading 14 s, search 1.5 s) for the same search. It should be noted, however, that, because the database is already available (loaded) on PhageCloud's website, 37 s is for the searching step and website responses (average value for 5 runs on 5 different days) whereas GSearch took 1.5 s for this same step. For comparison, Mash took 4 minutes to find the same two best matches.

Graph building for fungal genomes is slower compared to prokaryotic genomes, despite the smaller number of available fungal genomes (*n* = 9700), because the average fungal genome size, and thus k-mer and sketch space, are much larger (*k* = 21, *s* = 48 000 were used). It took 2.3 h on a 24-thread node to build the nucleotide level graph for these fungal genomes. Searching step was also slower due to the larger k-mer space. Accordingly, it took 3.13 min to request 50 neighbors for 50 query fungal genomes while Mash tool 4.4 min. Nonetheless, top 10 recall was still very high (∼99.4%) using MUMMER-based ANI as the reference in the comparison. For the amino acid level graph, the time for graph building was 0.61 h, shorter than the corresponding prokaryotic graph. Identifying 50 neighbors for 50 query fungal genomes at the amino-acid level took 1.24 min (Mash took 2.59 min) with similarly high top 5 and top 10 recall (99.7% and 98.5%, respectively) against the BLAST-based AAI. Note that the difference in run time will be more pronounced between Mash and GSearch with a larger number of fungal database genomes available in the future, as also exemplified above for the bacterial genomes.

### Comparing the three-step framework for classifying prokaryotes against GTDB-Tk

To evaluate the accuracy of classification/searching results based on the complete 3-step framework of GSearch, we compared the best neighbors found by GSearch with brute-force ANI (estimated by FastANI) and GTDB-Tk, a reference standard for microbial taxonomy classification that is phylogenetic-tree-based. The reader is reminded that GSearch does not assign taxonomy to query genomes itself; rather, it only identifies the best match and the ANI/AAI value of the query genome to the best match(es). The user is then responsible to transfer the taxonomy of the best match to the query genome, and we offered suggestions above on what thresholds to use, e.g. if AAI is above 65% to assign it to the same genus. Accordingly, the comparison to GTDB-Tk (as well as the other approaches mentioned above) focused on whether the same best match was found by the two approaches and if yes, then the search (or taxonomic assignment) was considered to be successful (or correct).

The overall running time to classify 1000 prokaryotic genomes of varied levels of taxonomic novelty against the GTDB database using different computing platforms is showed in Supplementary Table S5. On a 24-thread Linux node with Intel Xeon Gold 6226 CPU, GSearch took a total of 5.85 min while it took 19.49 min on an intel Core i7 laptop (2017 release) CPU personal laptop (6.02 min on the most recent ARM64 CPU laptop). Classifying 1000 genomes using GTDB-Tk took 5.91 h on the same Linux node with 24 threads (memory requirement was ∼328GB). We also evaluated the most recent GTDB-Tk v2.0 with MASH option, which took 76.2 min for the same task. We were not able to assess Dashing or BinDash for this analysis because they do not provide proteome (amino acid) level implementations.

In terms of classification accuracy, all query genomes that had a best match higher than 78% ANI against the GTDB database genomes (i.e. a match at the same or closely related species, 699 out of the total 1000 queries) were identically classified by GSearch, GTDB-Tk and FastANI (Supplementary Table S4 and Supplementary file S1, 100/699 are shown). Therefore, species-level classifications are highly consistent, further confirmed by Sourmash LCA methods (Supplementary file S4, 100/699 are shown), which performs genome k-mer classification using an ‘lowest common ancestor’ approach. Among the remaining 301 genomes that did not have same or closely related species-level matches, 266 of them (or 87.1%) had identical classifications between GSearch and GTDB-Tk at genus or family levels (Supplementary Table S4) but several inconsistencies were observed for 35/301 genomes (Figure 3e). Specifically, we noticed that for GTDB-Tk, which relies on RED values and tree topology, several genomes (*n* = 14) were still classified at the genus level even though the AAI value against the best database genome in these case was below 60% (typically, genomes assigned to the same genus show >65% AAI(54)), and some genomes (*n* = 16) were still classified at the family level but not at the genus level even though their best AAI value was above 65%. Similarly, several genomes (*n* = 9) were classified at the order level but not family level even though their best AAI value was above 52%. Therefore, high consistency was overall observed between GSearch and GTDB-Tk assignments, and the few differences noted were probably associated with contaminated (low quality) MAGs or taxonomic inconsistencies, which was challenging to assess further, and/or the peculiarities of each method. Since ProbMASH-ANI distance correlated well with BLAST-based AAI after transformation in the range of AAI values between 52% and 95%, the classification results were always consistent with AAI-based classification based on the abovementioned thresholds. For example, best matches with AAI ≥ 65% were classified in the same genus by GSearch and BLAST-based AAI, and best matches of 52% < AAI < 65% were typically classified in the same family (but sometimes different genus within the family).

### Database split for large genome databases

For large databases (for example, >100 million bacterial genomes), the graph building and requesting step could require a large amount of memory (due to much larger k-mer space) that is typically not available in a single computer node. We therefore provide a database split solution for such large databases. The average database building time on each node for each piece of the database after the splitting step scales linearly with increasing nodes/processors (Figure 2G) and requires much less memory (1/*n* total memory compared to when building in one node, where n is the number database pieces after splitting; for GTDB v207 nucleotide graph building and *n* = 5, it will be 28.3/5 = 5.66 GB). The searching time scales sub-linearly with increasing number of nodes (Figure 2H) but offers the advantage of a reduced memory footprint with respect to the single-node search. The top 10 best neighbors with the database split strategy were exactly the same as the non-splitting strategy (Supplementary file S2). Note that without multi-node support (e.g. run database build sequentially), database build time is nearly the same with the non-split strategy, but memory requirement is 1/*n* (e.g. for GTDB v207, 28.3 GB/5 = 5.66 GB at nucleotide level and 27.7 GB/5 = 5.54 GB at amino acid level), even though total request time will be longer (time*n in Figure 2h). However, since the request step is very fast relative to the graph building step, overall runtime is still small with the database split approach even for a relatively large number of genomes. Therefore, the database split strategy is especially useful when memory requirement is not satisfied on the host machine for large genome databases.

## Discussion

A popular way to assess genetic relatedness among genomes is ANI, which corresponds well to both 16S/18S rRNA gene identity and DNA-DNA hybridization values, the gold standards of fungal and prokaryotic taxonomies (13). However, the number of available microbial genomes has recently grown at such a speed that the all versus all search using traditional BLAST-based ANI or faster k-mer-based implementations has become intractable. Phylogenetic approaches based on quick (approximate) maximum likelihood algorithms and a handful of universal genes as implemented, for example, in GTDB-Tk could be faster than brute-force approaches but are often not precise and require a large amount of memory for the querying step (15) while the database building step could take several weeks of run time. Further, approaches to speed up the searching step that rely on k-medoid clustering to avoid all versus all comparisons could be sometimes trapped into local minima because of arbitrary partitioning of database genomes into clusters, a known limitation of these methods (18). GSearch effectively circumvents these limitations by combining new k-mer hashing-based probabilistic data structures for fast computation of genomic relatedness among genomes (i.e. ProbMinHash, SuperMinHash and/or SetSketch) with a graph based nearest neighbor search algorithm (HNSW). Accordingly, GSearch is at least an order of magnitude faster than alternative approaches (e.g. Mash, Dashing, Sourmash, GTDB-Tk) for the same tasks based on current datasets and will be even faster as the number of database genomes grows due to O(log(*N*)) complexity (versus linear complexity for these other approaches). We have shown that the speed advantage of GSearch will be even larger as the number of database genomes continues to grow. Therefore, GSearch solves an important challenge associated with the tasks to search and/or classify microbial genomes and will serve well these tasks for years to come.

To the best of our knowledge, no current tools have combined two sub-linear algorithms, and thus can efficiently search very large genome databases while maintaining high accuracy. Several sub-linear algorithms such as Sequence Bloom Tree or COBS (cross-over between an inverted similarity and Bloom filters) can do sequence to sequence (or genome to genome) search but do not provide ANI/AAI values, which are key parameters for microbial genome search and classification (Table 1), and these tools are generally less accurate than the ANI/AAI-based approach. GSearch can handle several million of microbial genomes on a small-to-average computer cluster, or even personal laptop (depending on the database size), since the database file size is proportional to the total number of genomes in the database for fixed sketch size and graph parameters, and generally rather small. Specifically, with one million prokaryotic genomes, the dumped database file size will be 5.9 GB*20 = 118 GB (currently, there are ∼60K in the GTDB database, creating a database file size of 5.9 GB). With the SetSketch option, database file size will be 0.5 GB for the GTDB database, 2.5 GB for the entire RefSeq genome databases (∼318K genomes), and 9.8 GB for one million genomes, and thus save on disk space, if needed, for a slight decrease in accuracy. Further, due to the nature of the HNSW graph, there is no need to build the entire database at once; rather, the database can be split into smaller pieces, as exemplified above and depending on the computational resources available. For a modern laptop with 16 GB memory, a database with one million species can be split into 10 pieces, so the dumped file for each piece will be 11.8 GB, which can be efficiently loaded into the 16GB memory. With this logic, a computing node with 24 threads and 256 GB of memory available can easily deal with 20 million bacterial database genomes, or billions of bacterial genomes if using SetSketch. This represents a substantial improvement compared to existing tools for the same purposes. This database split idea of HNSW, or other graph-based NNS libraries more generally, has been successfully applied to several industrial-level applications for other purposes (38,39).

We also want to point out that the size of the compressed sketches (database file) from SetSketch is comparable to other space efficient algorithms such as HyperLogLog (with *b* = 2, Setsketch asymptotically corresponds to HyperLogLog), ExtendedHyperLogLog (55), HyperLogLogLog (56) and UltraLogLog (57) (25% more space efficient than HyperLogLog). The Shannon entropy of SetSketch we implemented is $\frac{m}{{ln( 2 )ln( b )}}( {( {1 - \frac{1}{b}} ) + \smallint _0^1{{z}^{1/( {b - 1} )}}\frac{{( {1 - z} )ln( {1 - z} )}}{{zln( z )}}dz} )$, where m is the number of registers, b is the key parameter of SetSketch. For b = 1.001 that we used here, the entropy is 13.24 per register (divided by m). Therefore, compared to an uncompressed register size of 16 bits, there is still some room for improvement for SetSketch. Theoretically, an additional 17.25% reduction in space could be achieved but it would add implementation complexity. For any HyperLogLog-like algorithm (e.g. SetSketch, UltraLogLog), a theoretical limit for sketch size is $O( {{{\epsilon }^{ - 2}} + log( n )} )$, or slightly worse but easy to implement (58,59), where n is the number of elements/k-mers while ε is the error. This means that we cannot improve further without losing accuracy as database size grows unless we rely on database split strategy. SetSketch shares this property with HyperLogLog, and thus we cannot improve space further without theoretical breakthroughs.

Another distinguishing aspect of GSearch (tohnsw module) is the speed and flexibility in building reference databases. Users could build reference databases (graphs) for any number and type (e.g. microbial versus viral) of genomes. The high efficiency in building graphs allows users to also test and optimize the key parameters of the graph, the M (maximum allowed neighbors) and ef_construct (width of search for neighbor during building) parameters. For any given database size, M and ef_construct determine the quality of the graph and graph build speed. Small M and ef_construct may lead to frequent traps in local minima, and thus, low recall while large M and ef_construct may lead to slow speed without proportional improvement in recall (Supplementary Table S7). Therefore, there is a tradeoff between accuracy and speed that should be evaluated first. However, for most users this task would not be necessary because they will work with pre-built databases such as those provided here. Also, GSearch provides an option to add new genomes to the database without the need to rebuild the database from scratch, making it convenient and very fast to expand the current genome database.

GSearch could also be applied to whole metagenome search and identification of the most similar metagenomes in a series or a database because the relatedness among metagenomes can be estimated in a similar way to genomes using ProbMinHash as implemented, for example, in the HULK software (60). However, to allow for sketching of the much larger metagenome sequence data (compared to genomes) for building the HNSW graph, weighted k-mer (e.g. ProbMinHash) approaches require much larger memory than unweighted approaches. Probabilistic or approximate counting of k-mer abundances, such as the Count-Min sketch or a combination of Count-Min Sketch with HyperLogLog sketch (61), can be used instead for k-mer counting for metagenome search and/or when the computer memory is limited (62,63). Similarly, we could seamlessly replace ProbMinHash with another relatedness algorithm should such an algorithm becomes available and has advantages in terms of speed and/or precision in its locality sensitive hashing property (and thus estimation of Jaccard similarity). For example, the SuperMinHash option is provided for its high accuracy over traditional MinHash but not speed (43), or other newer implementations such as the One Permutation MinHash with Optimal Densification for its speed (one hash function, average case O(*n* + *m*)) and locality sensitive hashing property (via optimal densification or faster densification)(31,42) (Supplementary Table S1 and S2). Note that the bottom-K sketch implementation in Mash (19) or FracMinHash (25) in Sourmash (64) use just one hash function or a much smaller number of hash functions than classic MinHash, and thus lose the locality sensitive hashing property, which is essential for nearest neighbor search purposes (31,42,65). Further, BagMinHash (66) or DartMinHash (67) for weighted (but not normalized) Jaccard similarity can also be used in place of ProbMinHash (also LSH). Since the number of genomic distance computations is O(log(*N*)) in GSearch, the computational time for estimating genomic distance for a pair of genomes is not a major bottleneck in overall computational speed because log(*N*) is always a small number. Accuracy in the genomic distance estimate is relatively more important and the main reason that ProbMinHash is the default option in GSearch. Related to this, ANI as currently implemented, for instance, in FastANI is not appropriate for this function because it is not metric; that is, symmetry and triangle inequality properties do not hold (20). Similarly, mutation rates (or ANI) estimated by FracMinHash, CMash and related tools (e.g. Sourmash or skani-based calculated ANI) are also not metric (25,68,69). To solve this ‘metric’ problem, a norm adjusted proximity graph (NAPG) was proposed based on inner product, and shows improvements in terms of both speed and recall using non-metric distances (70). This could be another direction for further improving the speed and accuracy of GSearch in the future. The four options provided as part of GSearch to estimate Jaccard-like similarity are all metric, which ensures neighbor diversity when building the graph, and they are equally applicable to any microbial genome, including viral, prokaryotic and fungal genomes.

K-mer-based methods for genetic relatedness estimation such as ProbMinHash have lower accuracy between moderately-to-distantly related genomes compare to alignment-based tools (see Supplementary Note S2 and S4 for further discussion). Our empirical evaluation showed that this relatedness level, for nucleotide searches, is around 78% ANI, and 52% AAI for the amino-acid searches (e.g. Supplementary Figure S1 and S2). To circumvent this limitation, we designed a 3-step framework as part of GSearch to classify bacterial genomes that show different levels of novelty compared to the database genomes. This framework included a search at the universal gene level for deep-branching genomes that are novel at the phylum level (AAI < 52%), for which searching at the entire proteome level is less accurate. Note that we did not make suggestions for thresholds for (best-match) AAI values below 52% (or, in other words, classifications of novel query genomes at a level higher than the family level) because the AAI values are too overlapping between the order, class and phylum levels based on existing taxonomy (71). We refer the users to other publications that propose AAI thresholds for these higher ranks (18,54). Finally, we decided to not assign taxonomy within GSearch because such taxonomic assignments and/or thresholds might be challenging to establish for viruses and microbial eukaryotes (or such thresholds would likely differ from the prokaryotic thresholds), and GSearch is aimed to be a universal (genome search) tool, not only prokaryotic. Hence, the primarily objective of GSearch is to find the most related genomes among millions of database genomes and let the decision for taxonomic assignment, if this is a desirable output, to the user.

Recently, methods that employ k-mers that allow mismatches, that is, spaced k-mers, have shown promise in accurately estimating genomic relatedness even among distantly related genomes with gains in speed (72). To apply spaced k-mers to entire genomes, the recently developed ‘tensor sketch’ approaches could be explored in the future to simplify the distance computation for distantly related bacterial and viral genomes (73) instead of relying on the mentioned 3-step framework. In the meanwhile, the default ProbMinHash approach employed is highly efficiently and, importantly, can effectively deal with incomplete genomes or genomes of (drastically) different length, a known limitation of traditional Mash-based methods (28). Comparing genomes of different length is not uncommon, e.g. bacterial genome sizes can differ by more than ten-fold, as can be the case between MAGs of different level of completeness or when searching a short sequence (e.g. a bacteriophage genome) against a large genome collection (e.g. the whole viral genome database). Our own analysis showed that ProbMinHash is robust down to 50% completeness level (Supplementary Table S12), which is also the most commonly used standard for selecting MAGs of sufficient quality for further analysis and reporting (74). ProbMinHash is also robust for genomes with repeats or gene duplications (e.g. fungal genomes) due to the k-mer weighting step by weighted MinHash, a property not shared by simple MinHash implementations.

In general, the genome relatedness estimated, or best database matching genomes identified, by GSearch were highly consistent with BLAST-based ANI/AAI results or phylogenetic placement of the genomes using GTDB-Tk, particularly for query genomes with closely or moderately related genomes in the database, e.g. related at the species or genus levels (Supplementary file S1, Figure 3E). For more distantly related query genomes relative to database genomes, classification results of GSearch showed some differences with GTDB-Tk. These differences were not always possible to assess further for the most correct genome placement but could be due, at least partly, to the incompleteness and/or contamination of query or/and database genomes, which renders the resulting concatenated alignment of universal genes used by GTDB-Tk unreliable (74,75), as a few amino-acid positions per gene are used in the final alignment. In contrast, the AAI and ProbMinHash approaches should be more robust to changes of a small number of genes because the entire proteome is considered.

Graph-based NNS methods achieve good performance compared to tree based and locality-sensitive hashing (LSH) methods or space partitioning methods (40). Building a HNSW graph relies on proximity of the database elements. Thus, if the distances among database elements, in our case genomes, cannot be effectively estimated, the navigation of graph becomes less efficient (e.g. gets trapped in local minima). This is especially problematic for highly sparse/distantly related and diverse datasets, like the viral genome database, in which two phage genomes could often share very little genomic information (k-mers). Our results confirmed this expectation when we attempted to build a nucleotide-level graph for viral genomes. Hence, the amino acid level will be much more robust for viral genomes and is the recommended level to use. Finally, recent advancements in proximity graph building could further reduce database building time from O(N*log(N)) to O(N*c) (where c is a constant, independent of the number of genomes or *N*) and achieve better search performance than existing approximate proximity graph (76). This approach essentially reduces the number of points/genomes to be compared during graph building, and will be explored in future versions of GSearch to provide additional speed-up and/or robustness for graph database building. The HNSW graph, and graph-based K-NNS in general, can be further improved by adding shortcut edges and maintaining a dynamic list of candidates compared to a fixed list of candidates used by default (77). Graph reordering, a cache optimization that works by placing neighboring nodes in consecutive (or near-consecutive) memory locations, can also be applied to improve the speed of HNSW (78). It should be mentioned that in our HNSW Rust implementation, a memory map was also implemented, which will solve the memory limit problem when building large graph with billions of data points or genomes. Another direction for further improvement of GSearch could be the use of Graphics Processing Unit (GPU) instead of CPU because GPUs are more efficient in handling matrix computations and machine learning tasks (79). We will explore these options in future versions of GSearch.

Finally, GSearch provides a framework to create a new data structure: combining probabilistic data structures (e.g. MinHash, HyperLogLog) with a graph-based nearest neighbor search algorithm that should be applicable to not only genomes but also text searching purposes more broadly. For example, the MinHash algorithm or SimHash, another MinHash-like algorithm, (80) can be applied to document (e.g. website, text) similarity search when combined with HNSW. Further, we can hash strings to approximate edit distance (a metric distance) to avoid expensive dynamic programming (81) via order MinHash (82). In fact, we reimplemented order MinHash in our probminhash library, and it can be easily applied to do fast DNA string (not fragmented genomes) or general string search.

To summarize, GSearch, based on MinHash-like algorithms and HNSW, solves a major current challenge in search and classification of microbial genomes due to its efficiency and scalability. Both the Densified MinHash and HNSW are approximating the theoretical optimality of similar algorithms in terms of speed and accuracy trade-off. GSearch will serve the microbial sciences for years to come since it can be equally applied to fungal, bacterial, and viral genomes, and thus offer a common framework to identify, classify and study all microbial genomes at a million-to-billion scale.

## Supplementary Material

gkae609_Supplemental_File

## Data Availability

All the mentioned pre-built database for bacteria, fungi and phage genomes can be found at: http://enve-omics.ce.gatech.edu/data/gsearch. Code can be found here: https://github.com/jean-pierreBoth/gsearch or via Zenodo (https://doi.org/10.5281/zenodo.10543594) (83). GSearch relies on kmerutils v0.0.10 (https://github.com/jean-pierreBoth/kmerutils) (83), which is a Rust package that we developed to process genome fasta files including k-mer string compression, recursive k-mer hashing, k-mer counting and filtering using cuckoo filter. All tests performed in this study were based on GSearch version 0.1.3. Scripts for reproducing the results of this study can be found here: https://github.com/jianshu93/gsearch_analysis.
